# Gestational Diabetes Mellitus: What Can Medical Nutrition Therapy Do?

**DOI:** 10.3390/nu16081217

**Published:** 2024-04-19

**Authors:** Xiaoyi Wei, Hong Zou, Tingting Zhang, Yanling Huo, Jianzhong Yang, Zhi Wang, Yu Li, Jiuxiang Zhao

**Affiliations:** 1 CAS Engineering Laboratory for Nutrition, Shanghai Institute of Nutrition and Health, University of Chinese Academy of Sciences, Chinese Academy of Sciences, Shanghai 200031, China; weixiaoyi2022@sinh.ac.cn (X.W.); hzhou01@sinh.ac.cn (H.Z.); ttzhang@sinh.ac.cn (T.Z.); huoyanling2021@sinh.ac.cn (Y.H.); liyu@sinh.ac.cn (Y.L.); 2 Sunline Research Laboratories, Jiangsu Sunline Deep Sea Fishery Co., Ltd., Lianyungang 222042, China; y13814661888@163.com (J.Y.); laurent.wang@cmigroup.com.cn (Z.W.)

**Keywords:** gestational diabetes mellitus, medical nutrition treatment, nutrients, nutritional intervention

## Abstract

Gestational diabetes mellitus (GDM) is one of the common complications during pregnancy. Numerous studies have shown that GDM is associated with a series of adverse effects on both mothers and offspring. Due to the particularity of pregnancy, medical nutrition treatment is considered to be the first choice for the treatment of GDM. This contribution reviews the research progress of medical nutrition treatment in GDM, summarizes the international recommendations on the intake of various nutrients and the influence of nutrients on the prevalence of GDM, and the improvement effect of nutritional intervention on it, in order to provide references for research in related fields of GDM and the targeted development of enteral nutrition.

## 1. Introduction

Gestational diabetes mellitus (GDM) is one of the common metabolic disorders during pregnancy, which refers to various levels of carbohydrate intolerance first discovered during pregnancy [1]. Globally, the prevalence of GDM varies greatly by region, ranging from 1% to 31% [2]. The International Association of Diabetes and Pregnancy Study Group (IADPSG) recommends that all women who do not have diabetes during pregnancy should receive a 75 g oral glucose tolerance test (OGTT) at 24 to 28 weeks gestation. GDM is diagnosed when fasting/1 h/2 h plasma glucose levels exceed 92 mg/dL/180 mg/dL/153 mg/dL [3,4]. GDM is not only associated with a series of adverse pregnancy outcomes such as macrosomia, stillbirth, neonatal metabolic disorders, but also may increase the risk of diabetes and cardiovascular disease in the mother’s later years [5,6,7]. According to data from the International Diabetes Federation (IDF) in 2019, about 200,000 (16%) of newborns had hyperglycemia during pregnancy, 84% of which were caused by GDM. In addition, one in six newborns were affected by gestational diabetes after birth [8].

Medical nutrition therapy (MNT) means to prevent and treat diseases by regulating the nutritional status of the human body through the use of food and nutrients, which is an effective intervention for the management of chronic diseases such as diabetes [9]. Compared with drug therapy, MNT is safer and more economical and practical because of its natural nutrient sources and has become the first choice of GDM treatment [10]. Many studies have reported the dietary principles that people with GDM should abide by, but few studies have systematically summarized the application of MNT in people with GDM. Therefore, this paper will review the research on MNT in GDM, focusing on the study of MNT intervention in a GDM population and the influence of nutrient imbalance on GDM, in order to provide reference for the research in related fields of GDM, especially for the research and development of enteral nutrition.

## 2. Physiopathology of GDM

GDM is characterized by the inability of pancreatic β-cells to adequately respond to the increased demand for insulin during pregnancy, resulting in various degrees of hyperglycemia [1]. Similar to type 2 diabetes mellitus (T2DM), GDM is also a multifactorial disease whose pathogenesis is not fully understood. But, dysfunction of β-cells and failure of insulin secretion in response to pregnancy-induced insulin resistance have been shown to be likely key factors leading to GDM [11].

During a normal pregnancy, beta cells undergo hyperplasia and hypertrophy to meet the metabolic demands of pregnancy. During GDM, β-cells are unable to compensate for the demands of pregnancy and insulin sensitivity is reduced, leading to hyperglycemia. Most of the susceptibility genes associated with GDM are related to β-cell function, including potassium voltage-gated channel KQT-like 1 (Kcnq1) and glucokinase (Gck) [12]. On the other hand, glucose is the main source of energy for fetal growth. Studies have shown that insulin resistance will develop during pregnancy. Therefore, the cells will no longer respond adequately to insulin to limit the use of maternal glucose, and the adequate glucose supply will be diverted to the growing fetus [13]. Compared with a normal pregnancy, people with GDM have a 54% reduction in insulin-stimulated glucose uptake [14]. This insulin resistance is quite severe in the third trimester, approaching the level of resistance seen in non-pregnant people with T2DM [15]. It is worth noting that β-cells, blood sugar, and insulin sensitivity may return to normal after the end of pregnancy, but they may also continue to be impaired, thus evolving into T2DM.

## 3. Application of Medical Nutrition Therapy in GDM

**Figure 1:**
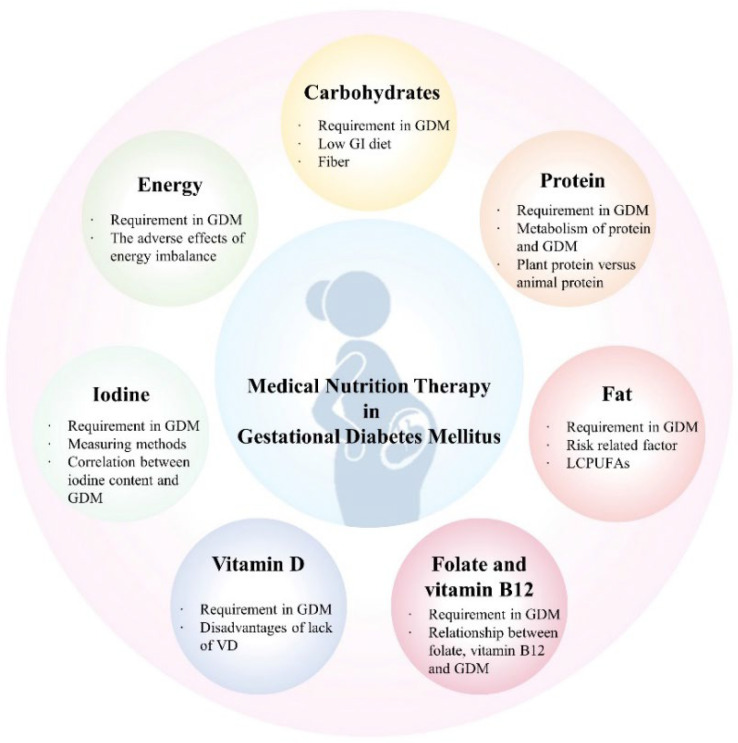
**Overview of medical nutrition therapy in gestational diabetes mellitus treatment**. GI, glycemic index; LCPUFAs, long-chain polyunsaturated fatty acids; GDM, gestational diabetes mellitus.

Dietary interventions have been widely recognized as effective in people with GDM. Overall improvements in women’s nutrition and health before and during pregnancy may contribute to fetal growth. Clinical studies have shown that the use of MNT in people with a diagnosis of GDM achieves the goal of maintaining glucose balance during pregnancy [16,17], improves insulin sensitivity [18], and reduces the risk of pregnancy complications [19,20]. For an unborn fetus, MNT reduces the production of reactive oxygen species (ROS) to limit oxidative damage, thereby reducing the occurrence of excessive oxidative stress and inflammation in the placenta [21]. What is more, MNT can improve neonatal excessive birth weight [10], and reduce the risk of maternal metabolic disorders such as hypertension after childbirth [22,23]. The nutrients of concern and their application in GDM are briefly summarized (Figure 1).

### 3.1. Restriction of Energy Intake

The energy intake during pregnancy should meet the physiological and metabolic needs of the pregnant woman and the fetus at the same time. In addition, it should also achieve and maintain blood sugar levels under the premise of gaining appropriate weight during pregnancy. There are no internationally recognized guidelines for energy intake specific to GDM. Given that women with or without GDM have similar nutritional needs during pregnancy, the general principles of energy intake and weight gain during pregnancy can be extrapolated to the people with GDM [24]. As the mother’s basal metabolic rate (BMR) increases during pregnancy, so does the need for energy. The more recognized standard for energy intake is the American Diabetes Association (ADA), which recommends a minimum intake of 1800 kcal per day for mothers, and an appropriate increase in energy intake during the second and third trimester of pregnancy to maintain normal weight gain and physiological metabolic needs [25]. In addition, due to the differences in the individual circumstances of pregnant women, it is further recommended that GDM patients should control their daily energy intake between 30 and 35 kcal/kg according to their body weight in the initiative of the International Federation of Gynecology and Obstetrics (FIGO) [26]. The dietary guidelines for GDM introduced in China in 2018 combined the above two programs with similar energy intake recommendations.

Excessive daily energy intake combined with commonly less physical activity during pregnancy can lead to excessive weight gain. Studies have shown that adjusting caloric intake to meet weight gain during pregnancy may be beneficial in reducing the mother’s blood sugar and insulin levels without producing abnormalities in other metabolic disorders [27]. Moderate caloric restriction can limit excessive maternal weight gain and may also be associated with a small reduction in fetal weight, which may reduce the birth of macrosomia to some extent. However, it should be noted that too low an energy intake diet has adverse effects on mothers. Severe low calorie intake during pregnancy (<1500 kcal/day) may induce maternal ketone production and is adverse to the growth and development of the fetus. It is not desirable to blindly pursue low energy intake [28].

### 3.2. Low GI and High Quality of Carbohydrates

Hyperglycemia is one of the most common adverse consequences in GDM. The main dietary factor affecting postprandial blood glucose concentration is carbohydrates. Quantity as well as quality of carbohydrates affect postprandial blood glucose response [24]. Limiting carbohydrate intake in GDM, especially simple carbohydrates, can reduce the postprandial hyperglycemia, fetal glucose exposure, as well as the fetal overgrowth [29]. Both the American College of Obstetricians and Gynecologists (ACOG) and the Academy of Nutrition and Dietetics recommend limiting carbohydrate intake to 35% to 45% of the total daily energy intake [4,30]. However, because of the differences in dietary structure between China and western countries, the dietary guidelines for GDM patients in China have increased the proportion of carbohydrate intake, suggesting that the daily intake account for 45~55% of the total energy.

The glycemic index (GI) of a food represents the effect of the carbohydrate portion of the food on blood sugar concentration after a meal compared to or compared to glucose [31]. As early as 1981, researchers have proposed that GI plays an important role in postprandial glucose levels [32]. With the development of scientific research, more and more studies have shown that a low GI diet can effectively improve blood sugar control [33,34,35,36,37]. A randomized trial showed that a low GI diet also had a significant positive effect on pregnancy weight gain and maternal glucose intolerance [38]. Moniek Looman and associates found that the quality of carbohydrates is closely related to the risk of GDM. Focusing on the intake of high quality and complex carbohydrates (such as foods high in dietary fiber) may reduce the risk of GDM [39]. Another meta-analysis also suggested that increasing fiber intake has a beneficial effect on blood sugar control, and a high-fiber diet improves responses such as blood sugar control, lipids, body weight, and inflammation [40]. In addition, based on the current evidence, a low GI diet is fairly safe in GDM and is not associated with adverse maternal pregnancy outcomes [41]. However, the research on the significant improvement of GDM incidence by a low GI diet is still insufficient, and more in-depth exploration is needed.

### 3.3. Good Dietary Protein

Protein plays a crucial role in bone development and nitrogen homeostasis. Protein deficiency can lead to severe malnutrition [42]. The ADA recommends that women with GDM should consume at least 71 g of protein daily during pregnancy to meet growth needs [43], which is comparable with the dietary recommendations in China. Many people with GDM may choose to reduce carbohydrates and increase protein intake to lower blood sugar. However, dietary protein is also an important regulator of glucose metabolism. Although high intake of dietary protein may have a positive effect on energy homeostasis by inducing satiety and possibly increasing energy expenditure, studies have found that a high-protein diet may affect glucose homeostasis by promoting insulin resistance and increasing glucose allogenesis [44]. Dietary protein may act as a precursor of gluconeogenesis to stimulate hexosamine biosynthesis or activate the mTOR signaling pathway, thus playing a crucial role in the pathogenesis of insulin resistance [45]. Studies have shown that replacing energy from carbohydrates by increasing total protein may be significantly associated with a higher risk of GDM [46].

What is more, the quality of protein also affects the risk of GDM. Wei Bao et al. [47] found that compared with plant protein, intake of more animal protein is associated with the increased risk of GDM, On the contrary, replacing animal protein with plant protein may reduce the risk of GDM. Weijia Wu et al. [48] investigated the relationship between dietary protein patterns and the risk of GDM in pregnant women in China. They also showed that women who consumed traditional animal protein patterns of red or white meat had a higher risk of GDM compared to plant-based protein diets. Partially replacinganimal protein with plant protein diet may help to ameliorate the adverse effects of GDM.

### 3.4. Dietary Fat

Fatty acids are of great importance in glucose homeostasis. An increase in plasma free fatty acids may lead to dose-dependent inhibition of insulin-stimulated glucose uptake, leading to increased insulin resistance [49]. Generally, it is recommended that fat intake should be account for 30~40% of total energy intake [4,24,26]. On account of the traditional cognitive restriction of dietary carbohydrate intake in GDM population, dietary fat intake is easy to increase, which may be more likely to lead to obesity [50]. Studies have shown that triglyceride levels are significantly higher in women with GDM compared to women without GDM [51]. However, Katherine Bowers et al. [52] found that there was no significant association between total fat intake and GDM risk, whereas increased intake of dietary cholesterol and animal fat was associated with a significant increase in GDM risk. The risk of GDM was lower when plant fat was substituted for carbohydrates as a percentage of energy compared to animal fat. Another study similarly found that increased dietary cholesterol intake was associated with an increased risk of GDM [53]. It seems that the key factor affecting the risk of GDM is the type of dietary fat consumed rather than the amount.

Studies have shown that *n*-3 long-chain polyunsaturated fatty acids (LCPUFAs) are beneficial for enhancing insulin action and glucose tolerance in animals and humans [54,55]. Docosahexaenoic acid (DHA) and eicosapentaenoic acid (EPA) are two of the most common unsaturated fatty acids in the *n*-3 family and are commonly found in deep-sea fish or fish oil. In addition, krill, a small marine crustacean found in the Antarctic Ocean, is getting attention as a source of healthy lipids [16]. Krill oil extracted from krill is rich in highly bioavailable *n*-3 LCPUFAs (especially DHA and EPA) [56]. It is worth noting that the *n*-3 LCPUFAs in krill oil mainly binds to phospholipids. Growing evidence shows that the phospholipid form of *n*-3 LCPUFAs is delivered to tissues more efficiently than the fish oil-rich triacylglycerol form [57]. What is more, studies have found that krill oil can have a certain improvement effect on triglycerides (TG), total cholesterol (TC), and low-density lipoprotein cholesterol (LDL-C) [58,59]. Recent studies have found that GDM is associated with changes in maternal *n*-3 fatty acid status and placental *n*-3 metabolism, having an impact on infant neurodevelopment and later brain health [60]. Supplementation of LCPUFAs such as DHA and EPA in pregnant women, especially in GDM, is of great significance.

### 3.5. Vitamin and Mineral Supplements

Maternal micronutrient imbalances may contribute to fetal stunting and chronic disease through direct effects on hormonal adaptation and epigenetic gene regulation. Current vitamin and mineral supplement recommendations for GDM are mainly based on dietary guidelines for pregnant women in various countries. Due to the variety of micronutrients, this article will introduce the research progress of several popular micronutrient interventions in GDM.

#### 3.5.1. Folate and Vitamin B_12_

Folate and vitamin B_12_ are two key nutrients in early pregnancy, which can be metabolically combined with each other and involved in DNA methylation and cellular metabolism during the one-carbon metabolism.

Folate, also called Vitamin B_9_, is a group of water-soluble B vitamins consisting of three sections, a pteridine ring, a para-aminobenzoate (p-ABA), and a tail of L-glutamate with one or more gamma connections [61]. Folate exists in different oxidation states, of which tetrahydrofolate (THF) is its most reductive form and acts as an essential coenzyme factor in many metabolic reactions. Folate is naturally found in beans, dark green vegetables, and other plants. Humans cannot synthesize folate itself, so dietary folate supplementation is required [62]. Folate comes in two supplemental forms, a natural form that is consumed from the foods mentioned above, and a synthetic form, folic acid, which is obtained from fortified foods or supplements. According to the dietary nutrient reference intake recommendation of Chinese residents, the recommended dietary folate equivalent (DFE) for an adult is 400 μg/d. What is more, the demand for folate during pregnancy has increased to 600 μg DFE to meet the fetus–placenta unit growth and maternal metabolic needs for pregnant women. Folate deficiency can hinder the development of red blood cells, resulting in megaloblastic anemia. During embryogenesis, the lack of folate tends to cause the formation of abnormal nerves, leading to the occurrence and development of neurodegenerative diseases such as anencephaly and spina bifida [63,64].

Vitamin B_12_ (VB_12_), also known as cobalamin, can be found in many products of animal origin such as meat, fish, dairy products, liver, and eggs [65]. Similar to folate, VB_12_ is also involved in carbon metabolism, driving DNA synthesis and energy metabolism processes [66]. Yong Ge and associates [67] identified that VB_12_-dependent molecules and metabolic pathways in human ileal epithelial cells (iEC) microtissue cultures by transcriptome, metabolome, and epigenome analysis, showing that VB_12_ not only promotes fatty acid and mitochondrial metabolism of human iEC, but it also is essential for maintaining the DNA methylation program of epithelial cells. The recommended daily intake of VB_12_ for adults is 2.4 μg/d, and it is recommended to increase by 0.5 μg/d during pregnancy. VB_12_ deficiency may lead to pernicious anemia and abnormal neurological complications, increasing the risk of cardiovascular disease and ischemic stroke. In pregnant women and newborns, VB_12_ can be transported to the fetus through the placenta to prevent megaloblastic anemia, DNA damage, and osteoporosis. The deficiency of VB_12_ in pregnancy is highly associated with adverse pregnancy outcomes and abnormal neurodevelopment in newborns [68,69,70,71].

Due to the low bioavailability of natural dietary folate, it is recommended to add supplement synthetic folic acid for additional folate nutritional fortification for specific populations such as perinatal women. Additional folate supplementation during pregnancy has reached a relatively uniform consensus in the population. We can obviously know from the previous content that VB_12_ and folate are important parts of the carbon cycle. However, the human body’s metabolic capacity for folate is limited, and blind supplementation of folate without corresponding increase in intake of VB_12_ can easily cause excess folate into the carbon cycle. The folate that is not timely metabolized will be accumulated in the long-term, which may pose a threat to health. A prospective cohort study found that daily folate supplementation during early pregnancy was associated with an increased risk of GDM. Women with a pre-pregnancy BMI of 25 kg/m^2^ or more who take folate supplements daily during the first trimester are at higher risk of developing GDM [72,73]. In addition to the effects of high folate intake on the mother, studies have found that folate supplementation affects normal DNA methylation in offspring. When the mother was given a high folate supplement, it increased the glucose intolerance and insulin resistance in the offspring of male mice fed a high-fat diet [74]. More importantly, studies have shown that higher levels of folate and lower levels of VB_12_ in maternal red blood cells may increase the risk of GDM, and the level of folate/VB_12_ in red blood cells of GDM group in early pregnancy is significantly higher than that of the group without GDM [75]. Another 2017 study evaluated blood samples from people who returned to the hospital for follow-up at 26 to 28 weeks gestation to analyze the association between B vitamins (folate, vitamins B_6_ and B_12_), homocysteine, and gestational diabetes in their plasma. The results showed that the combination of insufficient VB_12_ and high folate concentration was associated with a higher risk of GDM, suggesting that an imbalance of folate and VB_12_ may be the cause of glucose intolerance [76]. We should pay attention to the supplementation of VB_12_ while supplementing folate.

At present, the mechanism of the increased risk of GDM caused by the imbalance of folate and VB_12_ is not clear. However, it may be related to the carbon metabolism process that both of them participate in. When VB_12_ is insufficient, the relevant conversion process of folate in carbon metabolism is inhibited, thus blocking DNA/RNA synthesis. What is more important, the damage of mitochondrial DNA is related to the development of insulin resistance, resulting in the occurrence of GDM and other conditions [76,77]. But, additional research is needed to confirm these findings.

#### 3.5.2. Vitamin D

Vitamin D (VD), also known as calcitol, comes in two forms: ergocalciferol (D_2_) and cholecalciferol (D_3_) [78,79]. 7-Dehydrocholesterol in human skin can be directly synthesized into VD under ultraviolet irradiation through sunlight [80]. In addition, VD can be also naturally found in foods such as cod liver oil and egg yolks and can be supplemented through food. VD in food is consumed by the body and circulates to the liver in the blood, where it is converted to 25-hydroxyvitamin D (25(OH)D). Then, it will act in the active form of 25-dihydroxyvitamin D (1,25 dihydroxyvitamin D, 1,25(OH)_2_D_3_) [81,82]. According to the US Institute of Medicine, a serum 25(OH)D concentration below 50 nmol/L indicates insufficient VD intake, while a concentration below 25 nmol/L indicates a VD deficiency [83]. The recommended intake of VD for pregnant women is 10 μg/d, not different from that of the general adult population. However, studies have shown that VD deficiency is common at every stage of life, including pregnancy [84,85]. VD plays a crucial role in pregnancy and fetal development, which can maintain the immune regulatory environment required for normal pregnancy, prevent miscarriage, provide calcium for fetal bone development, and promote normal brain development [86].

Studies have shown that VD deficiency may be closely related to the occurrence of GDM and various adverse pregnancy outcomes [84]. Veronica T. Boyle et al. [87] investigated the association between concentrations of 25(OH)D in the first trimester and pregnancy outcomes such as GDM, preeclampsia, preterm birth, and small for gestational age (SGA). The results suggest that women with adequate VD intake (25(OH)D > 75 nmol/L) and women with lower VD intake (25(OH)D < 75 nmol/L) both have a higher risk of developing GDM during early pregnancy (OR 2.3; 95% CI 1.1, 5.1). But it’s not associated with the occurrence of pre-eclampsia, spontaneous preterm birth, or SGA infants. Meng-Xi Zhang et al. [88] conducted a meta-analysis based on a large number of studies to investigate the effect of maternal VD levels on GDM. The results showed that VD deficiency significantly increased the risk of GDM, and the higher the degree of deficiency, the greater the trend of GDM risk association. What is more, studies have shown that vitamin D supplementation alone for pregnant women may reduce the risk of preeclampsia, GDM, and severe postpartum bleeding [89].

The mechanism of the increased risk of GDM caused by VD deficiency has not been fully elucidated. However, VD plays a functional role in insulin secretion and maintenance of glucose metabolic homeostasis [90,91], which may be part of the reason for the increased prevalence of GDM caused by VD deficiency.

#### 3.5.3. Iodine

Iodine is an essential nutrient for the synthesis of thyroid hormone (TH) and is important for human growth and development [92,93]. Most foods and beverages are naturally low in iodine, while foods of marine origin have a higher iodine content due to ingestion of iodine-enriched seawater [94]. Iodine deficiency (ID) can lead to a series of adverse reactions, such as goiter, hypothyroidism, stillbirth, cretinism, and impaired cognitive development [95]. Dietary recommendations suggest that pregnant women consume 230 μg/d of iodine (120 μg/d for adults) to ensure normal maternal thyroid function and adequate thyroid hormone transfer to the fetus [96]. Since ID is very common, many areas use salt iodization for iodine fortification to reduce the harm caused by ID. However, ID is still very serious in pregnant women [97], and can lead to a series of adverse pregnancy outcomes.

Most dietary iodine is excreted into the urine within 24 h of ingestion, so 24-h urine iodine is considered the reference standard for measuring an individual’s daily iodine intake. Because of its simplicity, it is also the preferred and one of the most popular tests for assessing iodine status at the population level [98]. Based on the World Health Organization’s (WHO) iodine nutrition standards for pregnancy, Chenling Fan et al. [99] divided 1569 pregnant women into women with adequate iodine (UIC, 150~249 μg/L), mild iodine deficiency (UIC, 100~150 μg/L), moderate, and severe iodine deficiency (UIC < 100 μg/L), and more than sufficient and excess (UIC ≥ 250 μg/L) were found to have a higher risk of GDM. A 2020 prospective cohort study based in Rio de Janeiro, Brazil, found that insufficient and excessive iodine intake has an impact on pregnancy, and may impair adaptive mechanisms of maternal thyroid function, and contribute to the occurrence of GDM and adverse pregnancy outcomes [100]. It is worth noting that although urinary iodine is commonly used, it mainly reflects recent dietary iodine intake. Placental iodine load can better reflect the iodine status of women during long-term pregnancy, and it may be more representative of the iodine status of pregnant women [101,102,103]. Kristof Y. Neven et al. [104] measured the concentration of iodine in the placenta and found that higher concentrations of iodine in the placenta reduced the risk of GDM in the mother. Based on the above study results, we reasonably speculated that the iodine concentration in pregnant women may be somewhat correlated with the risk of GDM. However, since iodine deficiency and iodine excess are related to the occurrence of GDM, the correlation between iodine concentration and GDM risk may show a U-shaped result.

## 4. Conclusions and Future Perspectives

This article systematically reviewed the research progress of medical nutrition treatment in GDM, focusing on the relationship between the metabolism and intake of various nutrients and the risk of GDM during pregnancy, and the improvement of medical nutrition intervention on GDM (Table 1). Compared with previous analyses, this paper included more recent clinical studies while providing dietary recommendations and emphasizes the interaction between nutrients rather than the influence of a single nutrient on an individual (such as carbohydrate and protein, folate, and VB_12_), so as to provide certain practical guidance for dietary treatment of GDM.

More importantly, studies have shown that a combination of diet and exercise has positive implications for the treatment of GDM [105,106]. People with GDM are encouraged to take some physical exercise along with a healthy diet. Studies have found that women with a history of GDM are almost 10 times more likely to develop into T2DM than women with normal glycemic pregnancies [107]. It is urgent to improve this situation. At present, there are some enteral nutrition products developed for diabetic patients. However, there are no products about enteral nutrition for GDM. It is obvious that, compared to average adults, there are still some differences in the demand for various nutrients during pregnancy. It should not only meet the maternal nutritional requirement, but it is also necessary to consider the impact on the fetus. The research and development for individual-based treatment may become one of the future research trends. In addition, with the pursuit of sustainable development and healthy lifestyles, novel food ingredients, such as krill oil, may contribute to this field.

## Figures and Tables

**Table 1:** Summary of recommendations.

Nutrients	Recommendations
Energy	(1) A minimum of 1800 kcal per day is of great necessity, and should have an appropriate increase in energy intake during the second and third trimester of pregnancy. (2) Excessive and low daily energy intake are both undesirable.
Carbohydrates	(1) At least 35% to 45% of total daily energy intake is recommended, and it may be individualized in different countries. (2) A low GI diet is proved to be fairly safe in GDM and is not associated with adverse pregnancy outcomes.
Protein	(1) At least 71 g of protein daily during pregnancy should be ensured. (2) Replacing energy from carbohydrates by increasing total protein may be associated with a higher risk of GDM. (3) Eating more plant protein rather than animal protein may reduce the risk of GDM.
Fat	(1) Total fat intake should account for 30~40% of the total energy intake. (2) Compared to animal fat, the risk of developing GDM was lower when plant fat was substituted for carbohydrate as a percentage of energy. (3) Supplementation of LCPUFAs such as DHA and EPA in GDM is of great significance.
Folate and vitamin B_12_	(1) 600 μg/d DFE of folate and 2.9 μg/d of vitamin B_12_ is recommended. (2) Supplementation of VB_12_ should also be noted while supplementing folate.
Vitamin D	(1) 10 μg/d is recommended. (2) VD deficiency may be closely related to the occurrence of GDM and various adverse pregnancy outcomes.
Iodine	(1) 230 μg/d to ensure normal maternal thyroid function and adequate thyroid hormone transfer to the fetus. (2) Insufficient and excessive intake both have an impact on pregnancy.
